# Analysis of paint traces to determine the ship responsible for a collision

**DOI:** 10.1038/s41598-020-80088-5

**Published:** 2021-01-08

**Authors:** H. Lee, D. Lee, J. M. Seo

**Affiliations:** Korea Coast Guard Research Center, Korea Coast Guard Academy, Korea Coast Guard, Cheonan, 31254 Korea

**Keywords:** Ocean sciences, Characterization and analytical techniques

## Abstract

Although there have been many instances of ship collision at sea in recent times, not much research has been conducted on the topic. In this study, paint from an actual site of ship collision was collected and analyzed as evidence. The amount of evidence collected from the ships involved in the collision is either small or has inconsistent morphology. In addition, the contaminants and samples are often mixed in this evidence, making its analysis difficult. Paint traces of the damaged ship and the ship suspected to be responsible for the collision were compared through scanning electron microscopy with energy dispersive X-ray spectroscopy (SEM–EDS), attenuated total reflection–Fourier transform infrared spectroscopy (ATR–FTIR), thermogravimetry (TG) and derivative thermogravimetry (DTG), and pyrolysis–gas chromatography/mass spectrometry (Py–GC/MS) analyses. The ship responsible for the collision could be identified by characterization and by performing a comparative analysis of the extracted paint. Among the methods used in this study, Py–GC/MS can sensitively analyze even similar paints, and identified styrene and phthalic anhydride as the most prominent components of the paint used as evidence. The results obtained can be used to investigate the evidence collected from collision sites and to determine the ship responsible for the collision.

## Introduction

Increase in the global marine transportation has also increased the maritime traffic volume, leading to accidents at sea^1^. Marine accidents can be of various types such as ship collision, capsizing, grounding, and flooding^2^. Among them, ship collision is typically caused by inefficient operation, and may cause death, injury, and spillage of pollutants^3^. According to the Statistics Yearbook of Maritime Distress Accidents issued by the Korea Coast Guard in 2019, 372 collision accidents were reported in 2018^4^.

A vessel traffic service (VTS) system is used to identify the ships suspected to be involved in the collision. Based on the track recorded on the VTS, the operation record of the vessel passing through the sea area at the time of the collision is checked. Evidence must be collected to prove the damage caused to a ship in the case of a collision. Ship paint is an important and general physical evidence of a collision^5^. Paint can be collected from the body of each ship as evidence. Depending on the type of accident, we can find either little or no paint marks, or a large amount of paint, on the surface of a ship. If the impact area has only a few paint marks, it becomes difficult to collect the evidence because the common analysis methods cannot be applied. Therefore, an appropriate analysis method should be developed.

Analysis of marine paint material is essential to develop an appropriate paint analysis technology so that the authorities can quickly respond to marine accidents in the future. However, very few studies have been conducted on the topic, because it is difficult to collect paint material from the collision site as general public does not have easy access to ships involved in collisions. They also do not have the permission to collect samples. Recently, microplastics—a marine pollutant—has become an active research topic, and many studies have been performed to analyze the paint fallen off the ship surfaces^6,7^. Turner et al. analyzed antifouling paint particles obtained from abandoned structures and grounded ships using inductively coupled plasma-mass spectrometry (ICP-MS) and inductively coupled plasma optical emission spectrometry (ICP-OES). They reported that the paint chiefly comprised 35% Cu, 15% Zn, and Ba, Cd, Pd, Sn, and Ni trace metals^8^. Moreover, the elemental composition of marine paints is diverse. By identifying the constituent elements, the suspected ship can be identified.

Various techniques for the analysis of paints have been developed in the automotive field owing to the large number of road accidents or hit-and-run cases. In the case of a car accident, such as a ship collision, a thin layer of collision marks form, which is then analyzed. We believe that the techniques used for analyzing paints in the automotive sector can also be used for investigating marine accidents. Attenuated total reflection–Fourier transform infrared spectroscopy (ATR–FTIR) is one of the most common methods for automotive paint analysis^9–11^. However, if the paint contains similar polymer binders, more sensitive analysis methods are required^12^. The chemical components of paint can be identified through pyrolysis–gas chromatography/mass spectrometry (Py–GC/MS)^12–14^. The binder type can be identified even when the topcoat paint sample is only in micrograms, making it useful in criminal investigations^12^.

The paint used on ships is different from that used on cars. This is because the hull is immersed in sea water and is exposed to waves and strong ultraviolet rays. Malek et al. reported that alkyd paint, polyoxyethylene resin paint, phenolic paint, and amino alkyd paint are used to paint ship deck^15^. Almeida et al. described the typical paint systems used to paint the sides, superstructures, decks, tanks, and cargo of a ship^16^. Ship paint mainly comprises bisphenol A, toluene, benzene, and xylene^17,18^. Therefore, it is important to confirm the detection of these paint components, instead of analyzing the contaminants. The suspected ship was judged as the major offending ship based on blue samples collected from the same ship. Firstly, based on the analysis of the paint traces, the dominant offender was identified, and secondly, the facts of the accident were determined through investigations by the shipowner and the shipping company. This investigation is beyond the scope of our study.

In this study, the paint collected as evidence from three actual ship collision events occurring in 2020 was analyzed. Very few studies have investigated paint as the evidence of actual maritime accidents. Therefore, the aim of this study is to identify the offending ship through paint traces and to confirm the applicability of the collected data to similar accidents in the future.

## Materials and methods

### Sample preparation

**Figure 1:**
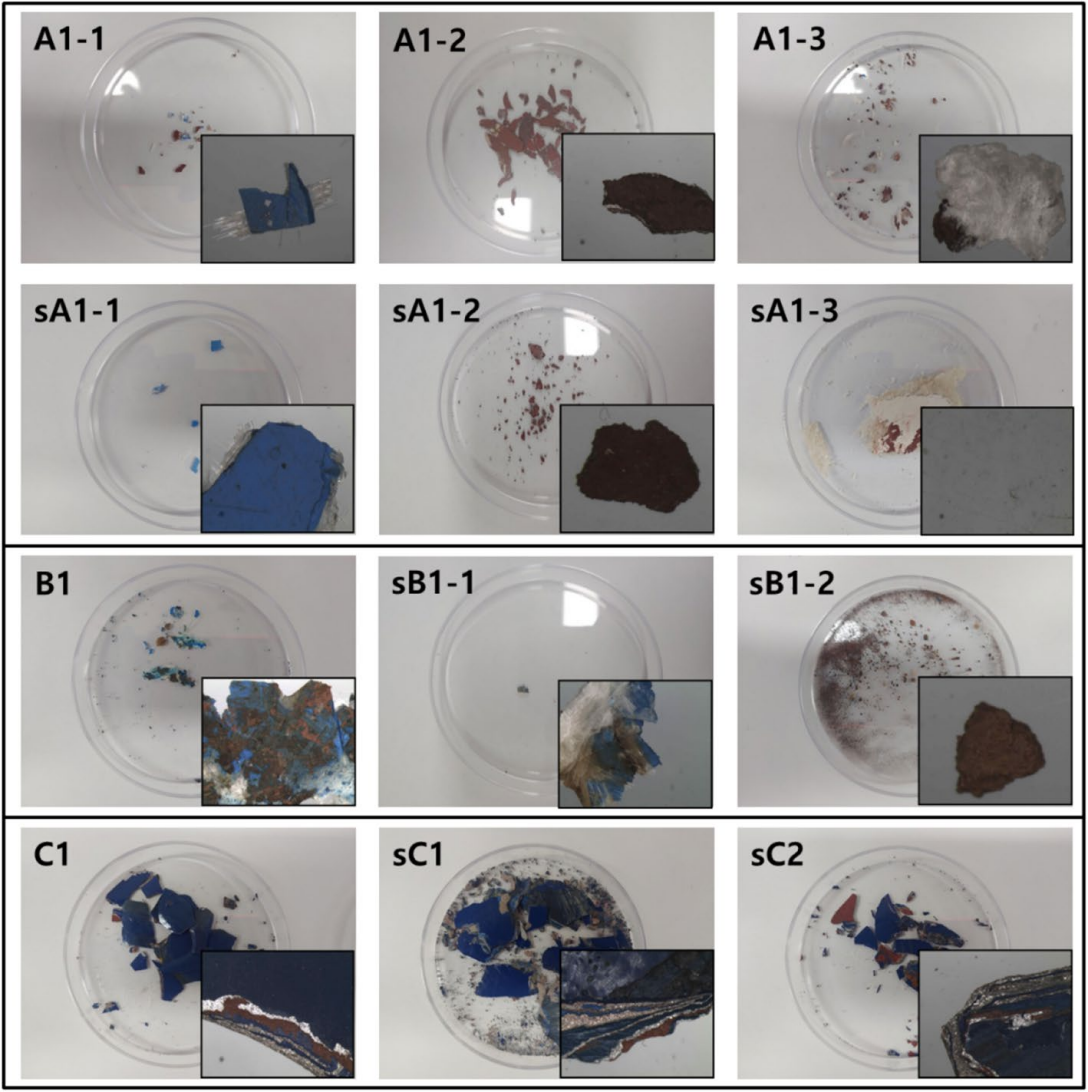
Paint collected from the actual collision site.

Paint samples were collected from three cases of ship collisions. Evidence samples were collected differently depending on the position and condition of the vessel. The samples were taken from various collision sites, including the deck, guardrails, port-side, and starboard. A representative sample was selected by comparing the paint colors. Table 1 lists information such as the accident date, ship color, and sample name. Figure 1 presents the state of the paint evidence. The bottom right of each image was magnified using an optical microscope. In Case I, the samples were taken from three collision sites of the damaged ship and the ship suspected to be involved in the collision. In Case II, one sample of a mixture of blue and brown paint was taken from the damaged ship and two samples from the suspected ship. In Case III, one sample was collected from the damaged ship and one sample each from the two suspected ships.

**Table 1:** Paint collected from the collision site.

	Accident date	Ship color	Sample	
			Damaged ship	Suspected ship
Case I	2020.06	Blue	A1-1	sA1-1
		White	A1-2	sA1-2
		Brown	A1-3	sA1-3
Case II	2020.03	Blue	B1-blue	sB1-1
		Brown	B1-brown	sB1-2
Case III (two suspected ships)	2020.03	Blue	C1	sC1
				sC2

### Analytical methods

To confirm the paint surface morphology and elemental composition, field emission scanning electron microscopy (FE-SEM) images were obtained using a JEOL JSM-7500F microscope (Tokyo, Japan) equipped with an x-act 6 EDS microanalysis detector (Oxford Instruments, United Kingdom). The images of the samples (5,000 × magnification) were obtained using an accelerating voltage (5 kV).

In situ attenuated total reflection-Fourier transform infrared (ATR‐FTIR) spectroscopy was performed using an FT/IR-6100 instrument (JASCO, Japan) with KBr disks to determine the paint binder type. The spectra were collected between 4000 and 650 cm^−1^. For each sample, the spectrum represented a collection of 40 scans at a spectral resolution of 4 cm^−1^.

In the present study, Py–GC/MS analysis was conducted to compare the properties of each sample using a Py-2020iD pyrolyzer (Frontier Lab, Japan) attached to a SHIMADZU 2010 gas chromatograph (Kyoto, Japan) coupled with a Shimadzu QP2010 Plus mass spectrometer (Kyoto, Japan). A paint sample was first placed in a metal sample cup and then in a 600 °C furnace with an interface of 350 °C under a helium atmosphere. The product gas was transferred to a metal capillary column (UA-5, 30 m × 0.25 mm id, 0.25 µm) for separation. The oven temperature program was set at 42 °C for 1.3 min, and then ramped at 5.5 °C/min to 325 °C. The compounds were identified by referring to the NIST 5th MS library.

Thermogravimetric (TG) analysis was performed to determine the thermal behavior of the paint using a TGA N-1000 analyzer (SINCO, Korea). To this end, 7 mg of the sample was nonisothermally heated from room temperature to 600 °C at a heating rate of 20 °C/min under nitrogen atmosphere.

## Results and discussion

### Case I: Ship collision in June 2020

**Figure 2:**
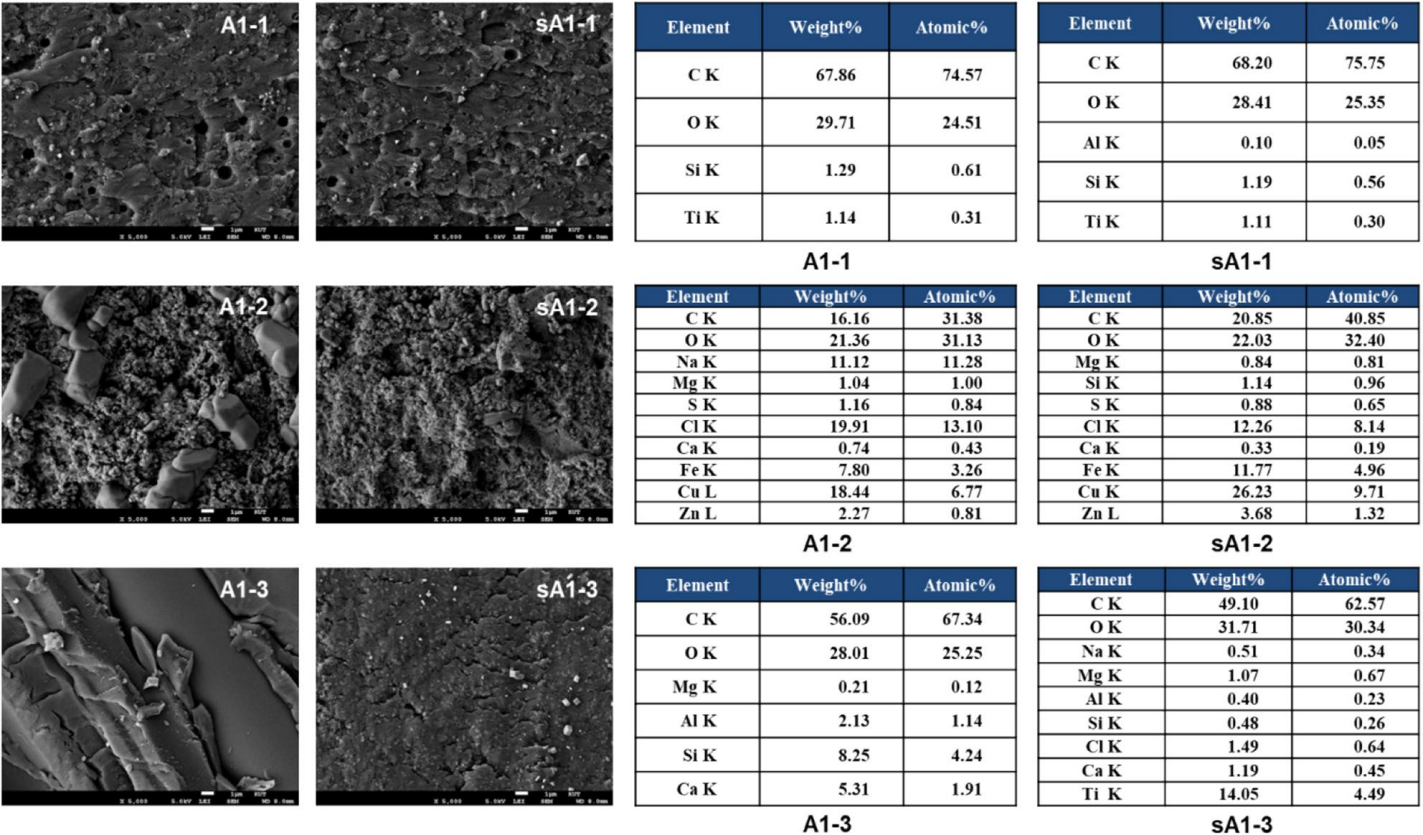
SEM–EDS image of Case I evidence.

Twelve samples were collected from the collision site. Of these, six were selected for a comparative analysis. The ship painted brown was the damaged ship, and the ship painted in white and blue was the suspected ship. The SEM surface images of the blue paint samples collected from the port of the damaged ship (A1-1) and the starboard deck of the suspected ship (sA1-1) are similar to each other (Fig. 2). In addition, the surface images of the brown traces of A1-2 and sA1-2 collected from the backside of the port and the part of the fore deck were similar. However, the surface images of the white paint collected from the port (A1-3) and starboard center (sA1-3) were different. C, O, Si, and Ti were observed in the blue fragments, while C, O, Cl, Fe, and Cu were detected as the main elements in the brown paint samples (EDS images, Fig. 2). However, more atomic elements were distributed in the white paint collected from the suspected ship than that collected from the damaged ship. In the damaged ship sample, C, O, Si, and Ca were detected as the principal elements, while Ti and Cl were also detected in the suspected ship sample. It is not possible to accurately determine why the analysis results of the white paint samples of the damaged ship and the offending ship are different. The analysis results may differ because of the contaminants or other traces. Optical microscopy and SEM analysis results are shown in Figs. 1 and 2, respectively. A fibrous material was observed in the white paint layer and a smooth long shape was observed in the surface morphology, suggesting that the paint may contain the fibrous material. Parks et al. and Soroldoni et al. reported the presence of C, O, Mg, Al, Si, S, K, Ca, Fe, Ti, Ba, and Mn, depending on the paint type^19,20^.

**Figure 3:**
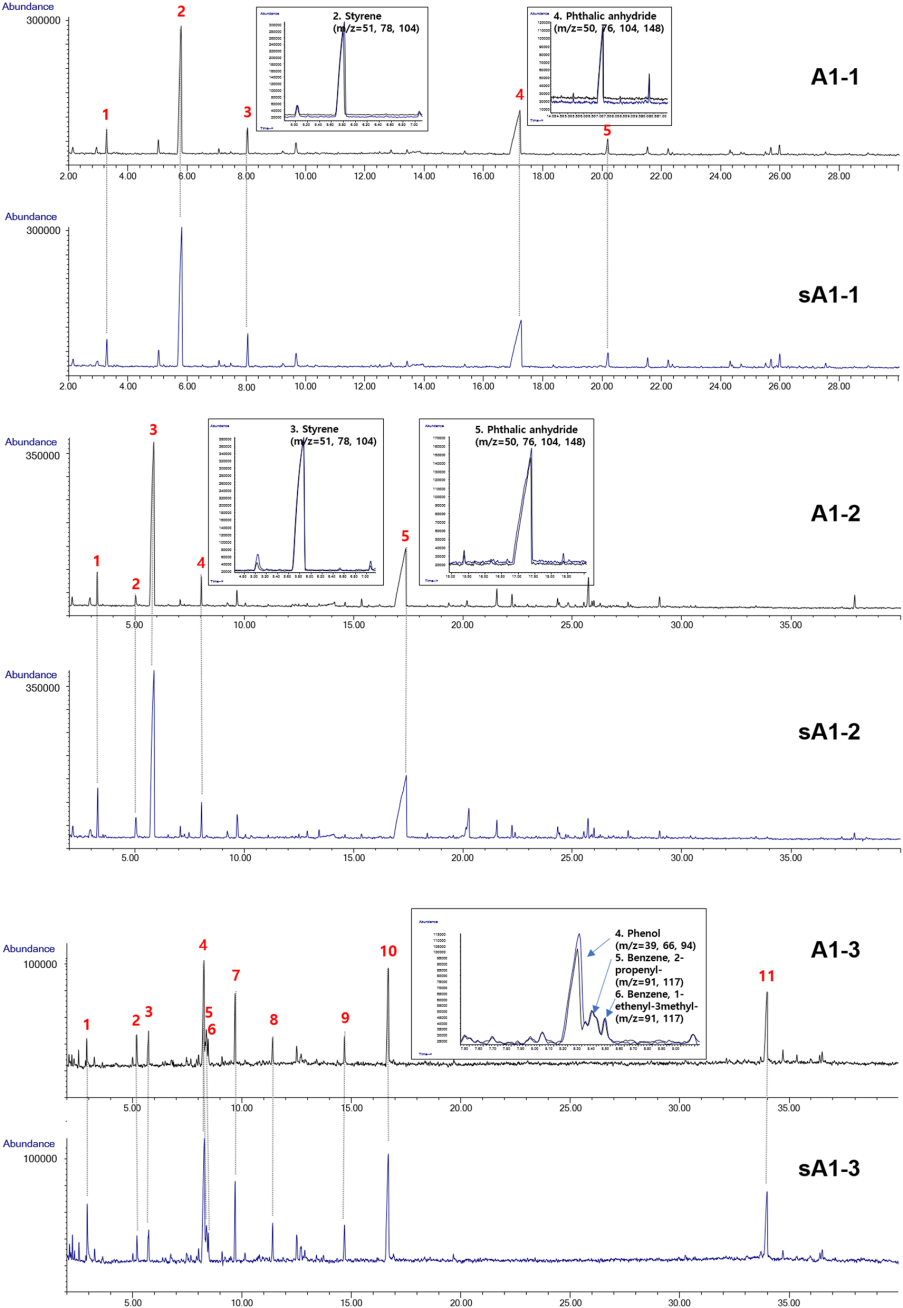
Py–GC/MS spectra of Case I evidence.

Figure 3 shows the chromatogram obtained from Py–GC/MS. The pyrolysis patterns of the blue, white, and brown samples of the damaged and suspected ships were similar. The major compounds of the blue samples were (1) toluene, (2) styrene, (3) α-methylstyrene, (4) phthalic anhydride, and (5) dimethyl phthalate. In the white paint sample, (1) toluene, (2) ethylbenzene, (3) styrene, (4) α-methylstyrene, and (5) phthalic anhydride were detected. In the brown samples, In the brown samples, (1) toluene, (2) ethylbenzene, (3) styrene, (4) α-methylstyrene, (5) phthalic anhydride (1) pyridine, (2) p-xylene, (3) p-xylene, (4) phenol, (5) benzene, 2-propenyl, (6) benzene, 1-ethenyl-3-methyl-, (7) indene, (8) triisopropylsilanol, (9) phenol, 4-(1-methylethyl)-, (10) p-isopropenylphenol, and (11) phenol, 4,4′-(1-methylethylidene)bis-were identified. Styrene and phthalic anhydride were the most prominent components. Phthalic anhydride is known to be dominant in alkyd formulations^21,22^. Poly(acrylate/styrene) is an unsaturated polyester widely used in emulsion-based paints^23–25^. Song et al. indicated that alkyds and poly(acrylate/styrene) derived from marine paint resins in microplastics dominate the sea surrounding Korea^23^. Pyridine and triisopropylsilanol were the characteristic compounds in the brown paint traces. These chemicals are used in antifouling paints for ships because they inhibit the growth of marine bacteria^26^. In addition, common ship surface-coating materials such as toluene, ethylbenzene, and xylene were identified.

The SEM–EDS analysis of the white paint traces taken from the damaged and suspected ships did not match. However, the surface elements of the other samples were similar, and the pyrogram patterns and compounds of Py–GC/MS were also consistent. It is not easy to match all the evidence collected from the site of maritime accidents, because the surface of the ship contains various contaminants and sampling is not easy. The surface morphology of the white paint did not match, but the blue and brown paint samples taken together perfectly matched. Therefore, it can be concluded that the suspect ship was the dominant offender.

### Case II: Ship collision in March 2020

**Figure 4:**
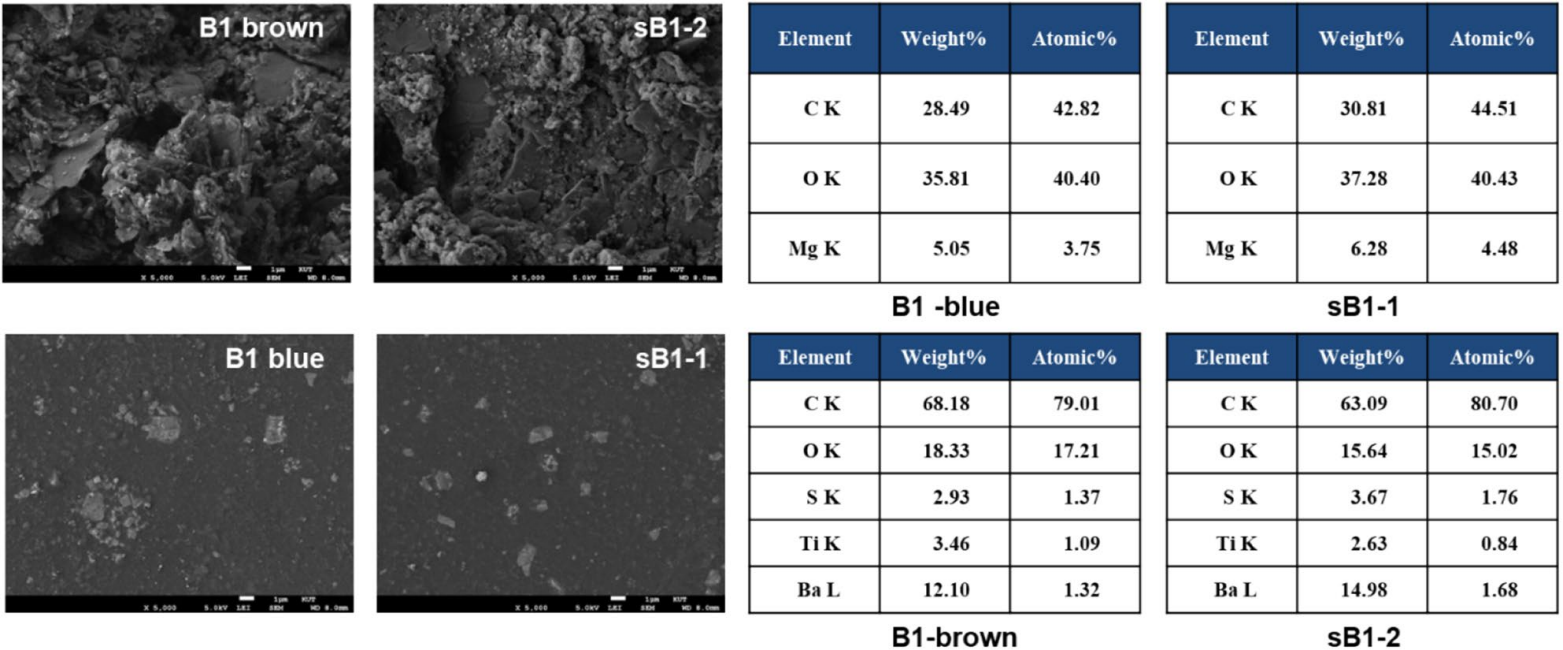
SEM–EDS image of Case II evidence.

Five paint traces were collected from the collision site, the paint color was visually checked, and then three representative samples were selected for this study. The traces were not large, and some samples were collected by scraping the ship guardrail, causing the paint traces to shred like powder. In addition, in some of the samples, the paint traces of both the damaged and suspected ships were mixed; therefore, it is necessary to check the paint colors carefully and separate them using an optical microscope. The brown and blue parts of the B1 sample were compared with the sB1-1 (blue) and sB1-2 (brown) samples, and it was observed that these samples had similar surface morphology (Fig. 4). The blue traces had a relatively smooth surface, while the brown samples had amorphous particles on the surface. C, O, and Mg in the blue samples and C, O, S, Ti, and Ba in the brown samples were detected by EDS analysis (Fig. 4).

**Figure 5:**
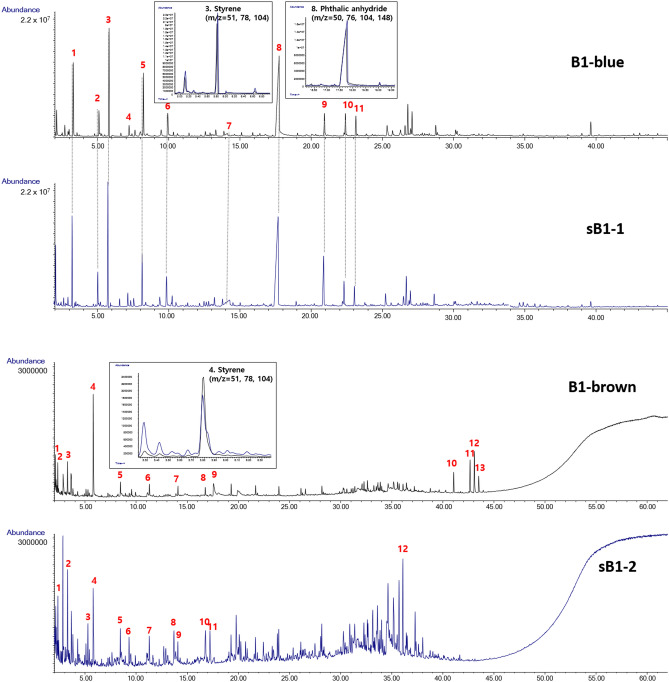
Py–GC/MS spectra of Case II evidence.

Figure 5 presents the Py–GC/MS results. The chromatograms of the blue samples, B1 and sB1-1, were similar. However, the brown samples of the damaged and suspected ships showed different patterns. The main components of the blue sample were (1) toluene, (2) ethylbenzene, (3) styrene, (4) 2-propenoic acid, 2-methyl-, 2-methylpropyl ester, (5) α-methylstyrene, (6) benzene, (1-methylenepropyl)-, (7) benzenecarboxylic acid, (8) phthalic anhydride, (9) dimethyl phthalate, (10) benzene, 1,1′-(1,2-ethanediyl)bis-, and (11) benzene, 1,1′-(1,2-ethanediyl)bis-. (1) benzene, (2) 1-heptene, (3) toluene, (4) styrene, (5) 1-decene, (6) 1-dodecene, (7) 1-dodecene, (8) 1-tridecene, (9) phthalic anhydride, (10) bis(2-ethylhexyl) phthalate, (11) phosphoric acid, tris(3-methylphenyl) ester, (12) phosphoric acid, tris(3-methylphenyl) ester, and (13) phosphoric acid, tris(3-methylphenyl) ester were identified in the B1 sample, and (1) benzene, (2) toluene, (3) o-xylene, (4) styrene (5) 1-decene, (6) benzene, 1-methyl-3-(1-methylethyl)-, (7) 1-dodecene, (8) naphthalene, (9) 1-dodecene, (10) naphthalene, 1-methyl-, (11) naphthalene, 1-methyl-, and (12) phenanthrene, 1-methyl-7-(1-methylethyl)- were detected in the sB1-2 sample. In the blue sample (B1 and sB1-1) as in Case I, phthalic anhydride, the main component of the alkyd paint, and styrene were observed. As shown in Fig. 5, the two samples showed similar peaks. Phosphoric acid, tris(3-methylphenyl) ester—a characteristic component of brown paint—was detected in B1, while naphthalene compounds such as (1) naphthalene, (2) naphthalene, 1-methyl-, and (3) phenanthrene, 1-methyl-7-(1-methylethyl)- were identified in sB1-2. The brown part was collected in a powder form from the ship guardrail, making it difficult to distinguish between the paint and the contaminants. In fact, it was difficult to identify the composition of a part of the sample because of the overall low intensity of the chromatogram.

The SEM–EDS analysis results of the damaged and suspected ships were similar, and the peak patterns and composition of Py–GC/MS the brown sample did not match. The suspected ship was determined to be the major offending ship based on the analysis of the blue samples collected from the same ship.

### Case III: Ship collision in March 2020

**Figure 6:**
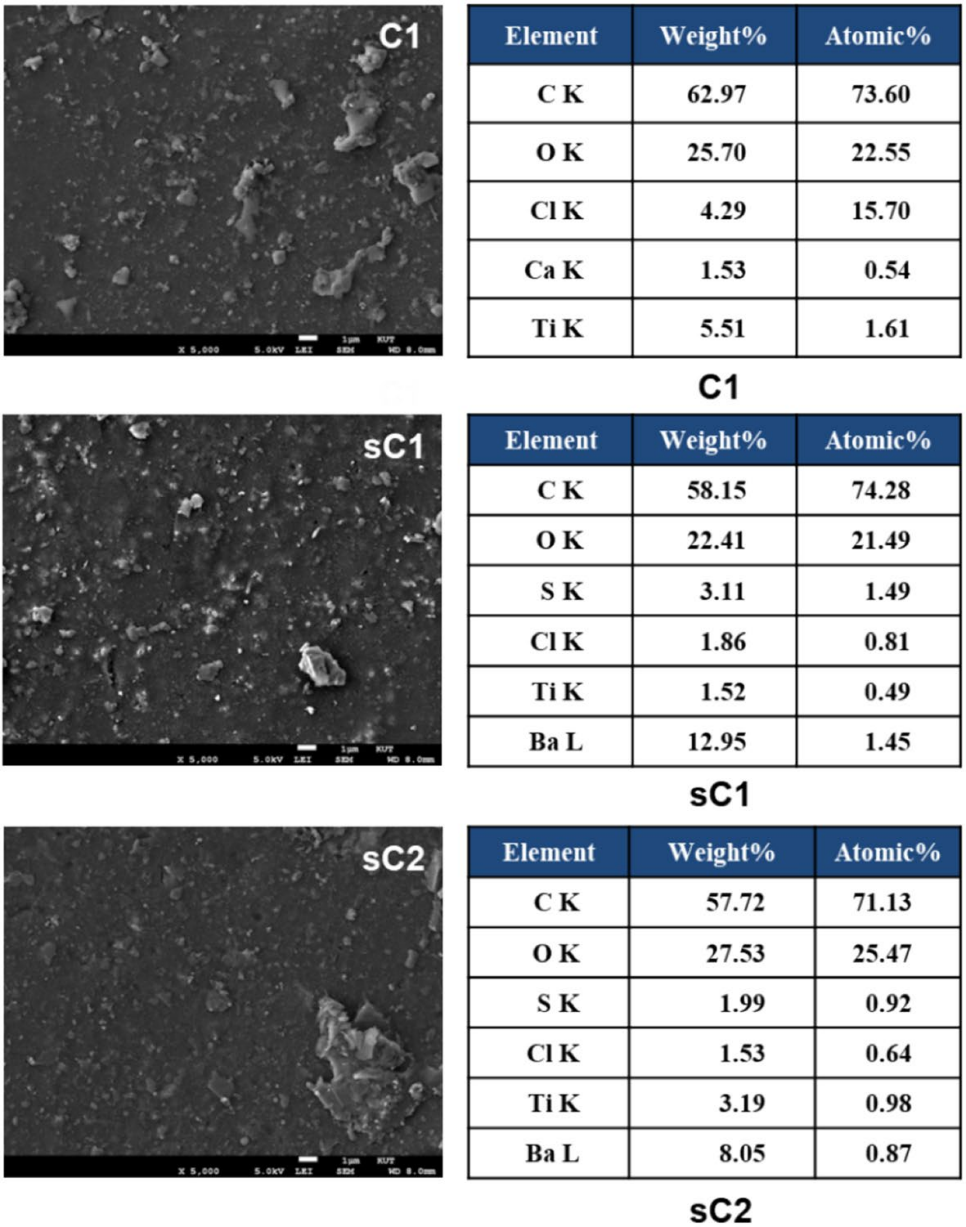
SEM–EDS image of Case III evidence.

Three samples were collected from the collision site. Two suspected ships were identified after investigating the damage. The damaged ship sample C1 and the suspected ship samples sC1 and sC2 had similar surface textures. However, the elemental analysis of the suspected ship samples conducted using EDS was not consistent with that of the damaged ship (Fig. 6). C, O, Ti, Cl, and Ca elements were detected in the damaged ship sample; S and Ba were also identified in the paint traces obtained from the two suspected ships.

**Figure 7:**
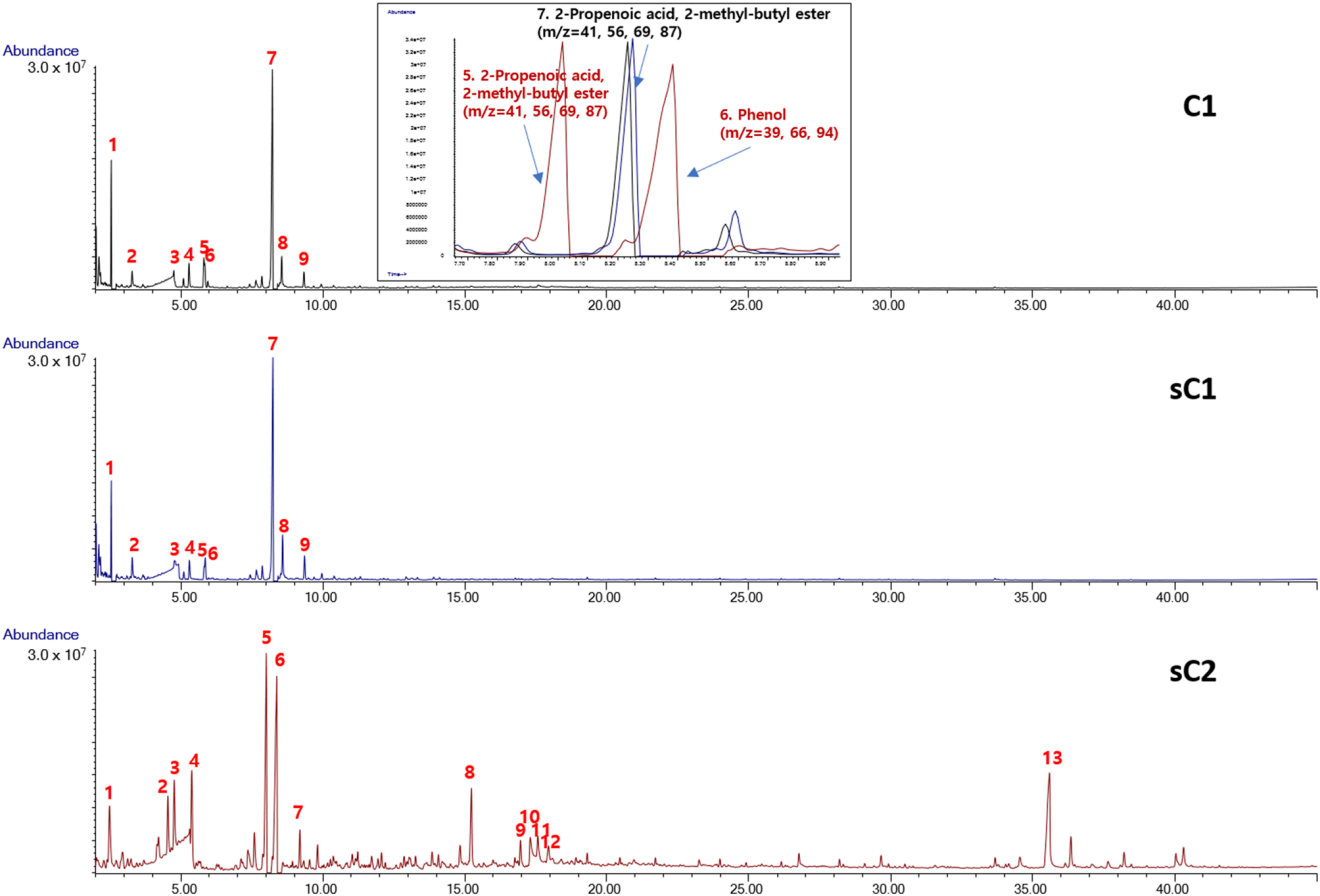
Py–GC/MS spectra of Case III evidence.

The chromatogram results of C1, sC1, and sC2 samples are shown in Fig. 7. C1 and sC1 showed similar chromatogram patterns, while sC2 showed a different profile. The following were the major compounds of C1 and sC1 samples: (1) 2-propenoic acid, 2-methyl-, methyl ester, (2) toluene, (3) 2-propenoic acid, 2-methyl-, (4) p-xylene, (5) styrene, (6) o-xylene, (7) 2-propenoic acid, 2-methyl-, butyl ester, (8) benzene, 1,2,3-trimethyl-, and (9) benzene, 1,2,3-trimethyl-. For the sC2 fragments, the major elements were (1) toluene, (2) ethylbenzene, (3) p-xylene, (4) p-xylene, (5) 2-propenoic acid, 2-methyl-, butyl ester, (6) phenol, (7) benzene, 1,2,3-trimethyl-, (8) phenol, 4-(1-methylethyl)-, (9) phenol, p-tert-butyl-, (10) p-isopropenylphenol, (11) phthalic anhydride, (12) 1,3-benzenedicarbonitrile, and (13) phenol, 4,4′-(1-methylethylidene)bis-. Hydrocarbons such as toluene and xylene were mainly detected in the C1 and sC1 samples. In the sC2 sample, in addition to hydrocarbons, O-compounds such as 2-propenoic acid, 2-methyl-, butyl ester, phenol, 4-(1-methylethyl)-, benzaldehyde, 3-methyl-, and N-compounds of 1,3-benzeneicarbonitrile were detected.

**Figure 8:**
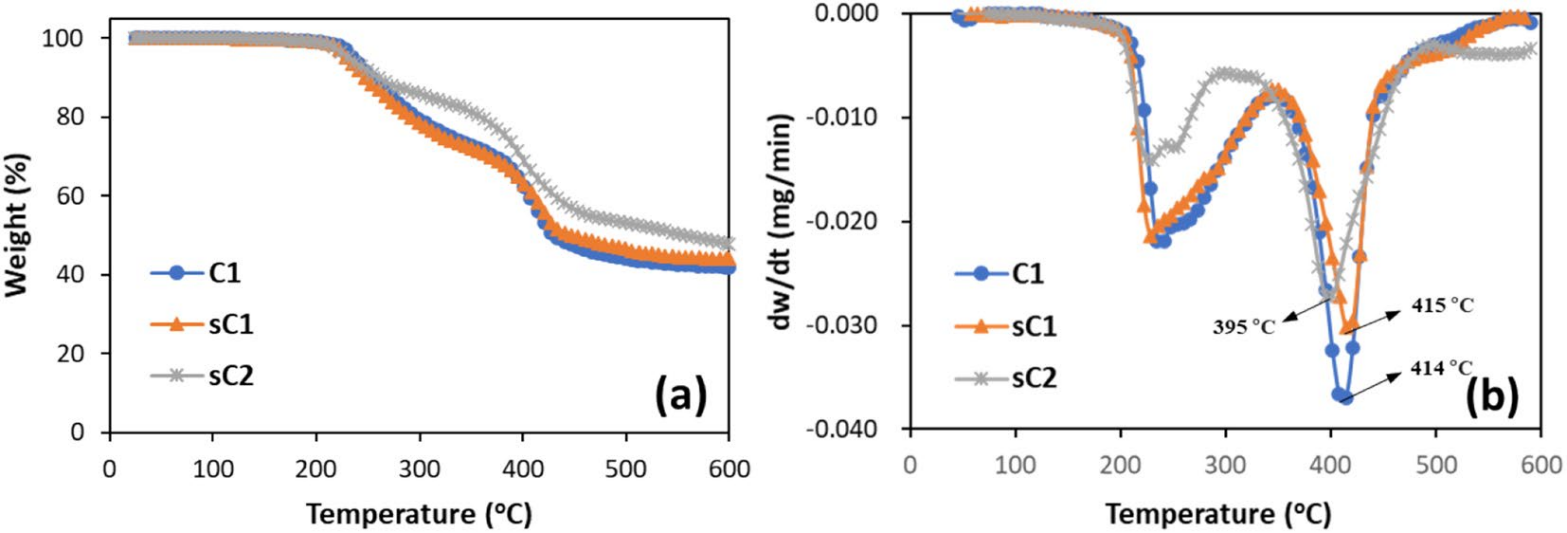
TG–DTG curves of Case III evidence.

Figure 8 shows the TG and DTG curves of the Case III evidence. All three samples present two decomposition stages in the TG and DTG curves. The first stage was from 200 to 350 °C, and the second stage was from 350 to 500 °C. The C1 and sC1 samples presented similar behavior; in contrast, sC2 showed less weight loss compared to the other samples. The weight losses of C1, sC1, and sC2 were 27%, 28%, and 19% at 350 °C, respectively, and 56%, 53%, and 47% at 500 °C, respectively. Because of the presence of phenolic compounds such as phenol, 4-(1-methylethyl)-, phenol, p-tert-butyl-, p-isopropenylphenol, phenol, 4,4′-(1-methylethylidene)bis in the sC2 sample, the weight loss was estimated to be less in the TG–DTG curve measured from room temperature to 600 °C. Zhou et al. and Souza et al. reported the thermal decomposition of a phenolic resin converted to an amorphous carbon at 700 °C, and showed that the thermogravimetric loss rate of pure phenolic resin reached a maximum at 550 °C^27,28^.

Few studies have used TG and DTG curves for paint analysis. The material components of the paint cannot be identified by TG analysis, and the analysis results obtained by weight loss only are not reliable. In addition to traces owing to collision, ship paint can acquire different traces during its voyage. It is often impossible to distinguish the two traces. Therefore, it is necessary to compare and analyze many samples. TG analysis can be used to reduce the number of samples prior to component analysis, because it then makes it possible to distinguish the suspected vessels even though there is a weight loss.

**Figure 9:**
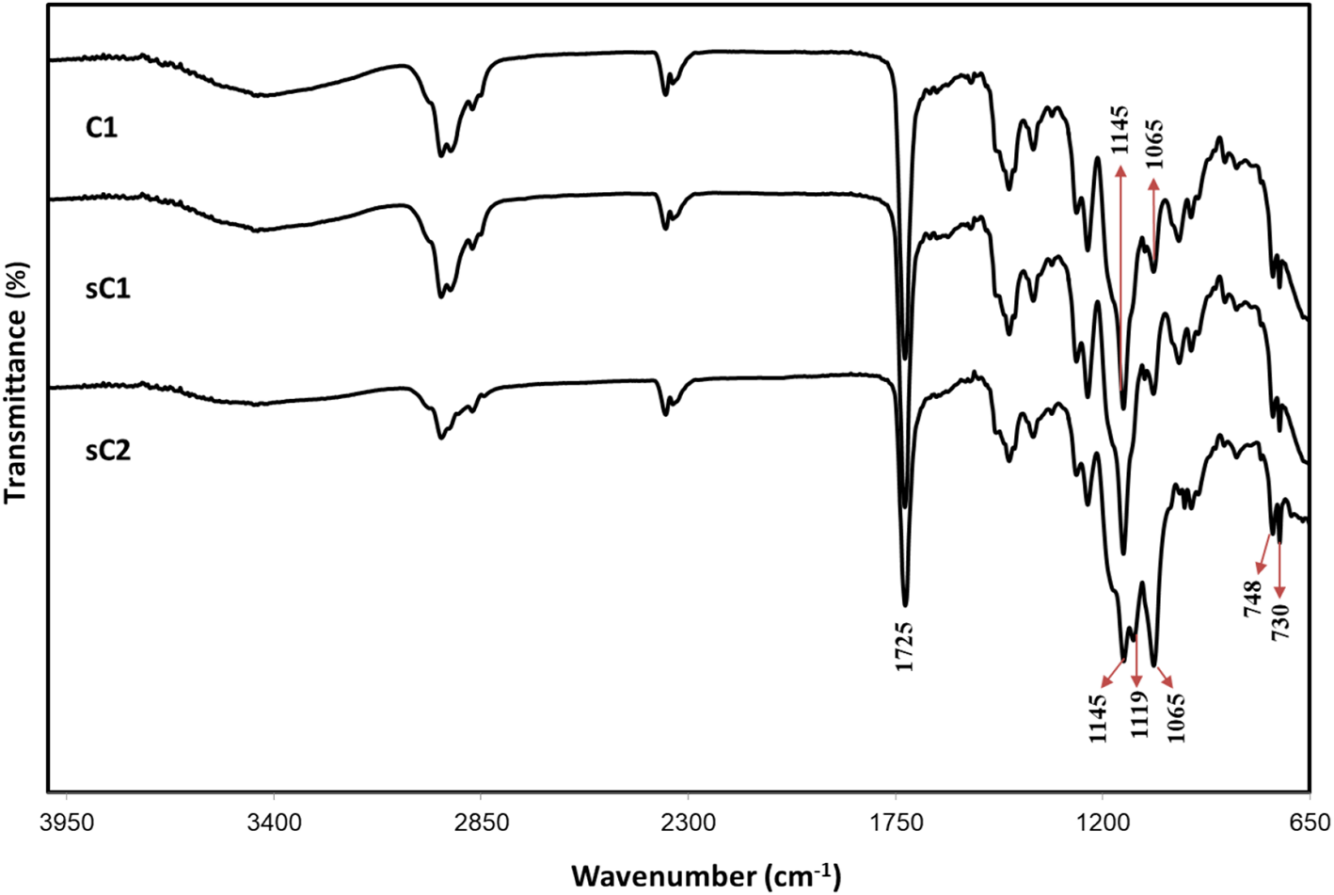
ATR–FTIR spectra of Case III evidence.

The infrared spectra of the Case III evidence are shown in Fig. 9. The ATR–FTIR spectra of C1, sC1, and sC2 showed similar absorption bands. In particular, the peak position and intensity of C1 were similar to those of sC1. An investigation of the paint evidence based on the spectra obtained from the FTIR polymer library at OMNIC (Thermo Nicolet Corporation, Sprouse Polymer by ATR Library) showed that the spectra matched well with that of poly(butyl methacrylate). A comparison of the paint evidence spectra with the Py–GC/MS results showed the presence of 2-propenoic acid, 2-methyl-, butyl ester (= butyl methacrylate), which showed the highest intensity in the chromatogram. These spectra are similar to those obtained for the alkyd resin, as reported by Hayes et al. and Burns et al. using ATR–FTIR. The spectral regions of the alkyd resin were identified as 1680–1770, 1000–1360, and 630–800 cm^−1^ for ester C=O, ester C–O–C, aromatic C-H and aromatic ring bending, respectively^21,22^. Although the spectra of the sC2 sample were similar to those of the damaged ship sample, different peaks were observed in the 1119 and 1065 cm^−1^ regions. He et al. analyzed the ship deck paint and reported peaks at 1120, 1070, 830, and 740 cm^−1^, indicating the presence of nitrocellulose and alkyd resins^5^.

Table 2 summarizes the results of SEM–EDS, Py–GC/MS, TG–DTG, and ATR–FTIR analysis. The paint samples had similar primary components, thermogravimetric decomposition curves, and binder type. Although SEM–EDS was measured at various locations of all the samples, particular elements such as Ba were observed in the suspected ships only. Although sC1 is a prime suspected ship, some results were inconsistent, and further analysis was not possible because the sample was taken from only one part of the ship. Because of the insufficient evidence, the ship cannot be judged as the offender. Therefore, it is important to collect several samples from the field to obtain accurate evidence.

**Table 2:** Summary of the results of Case III evidence.

Analyzer	Propose	Damaged ship (C1)	Suspected ship (sC1)	Suspected ship (sC2)
SEM–EDS	Specific elements, morphology	C, O, Cl, Ca, Ti	Not match	Not match
			C, O, S, Cl, Ti, Ba	C, O, S, Cl, Ti, Ba
PY–GC/MS	Compounds	Major compound 2-Propenoic acid, 2-methyl-, butyl ester	Match	Not match
			2-Propenoic acid, 2-methyl-, butyl ester	2-Propenoic acid, 2-methyl-, butyl ester, phenol
TG–DTG	Thermal behavior	Weight loss 27% at 350 °C 56% at 500 °C	Match	Not match
			28% at 350 °C 53% at 500 °C	19% at 350 °C 47% at 500 °C
ATR–FTIR	Binder type	Alkyd resins (poly(butyl methacrylate))	Match	Partial match
			Alkyd resins	Nitrocellulose, alkyd resins

## Conclusion

Paint, found as traces during a ship collision, is collected differently depending on the size and type of the accident. In general, evidence is collected after first visually inspecting the ship color. In this study, we used samples from three actual collisions. The collected paint evidence was analyzed using SEM–EDS, ATR–FTIR, TG–DTG, and Py–GC/MS. The surface morphology, constituent elements, and weight loss were confirmed through SEM–EDS and TG–DTG analysis of the paint traces, and the offending ship could be inferred. In addition, the paint surface materials were identified through ATR–FTIR spectra, and the paint components were analyzed using Py–GC/MS. Through this analysis, the offending ship could be identified, and the same trace analysis method can be used for investigating ship collisions in the future. Although the method described herein can be used for the analysis of paint on the surface of a ship, correct sampling is also important. Other traces and contaminants present on the ship surface often get mixed with the collision traces, making the analysis difficult. Therefore, it is necessary to collect as many collision traces as possible from the accident site and compare and analyze them all to correctly identify the offending ship.
